# Microscopic Mechanical Properties and Physicochemical Changes of Cement Paste Exposed to Elevated Temperatures and Subsequent Rehydration

**DOI:** 10.3390/ma18051050

**Published:** 2025-02-27

**Authors:** Lei Xu, Xiaochuan Hu, Ruifeng Tang, Xin Zhang, Yan Xia, Bo Ran, Jinlong Liu, Shiyu Zhuang, Weichen Tian

**Affiliations:** 1 Beijing Building Materials Academy of Sciences Research, Beijing 100041, China; lei.xu@epfl.ch (L.X.); tangruifeng@bbma.com.cn (R.T.); didazhangxin@163.com (X.Z.); 2 Laboratory of Construction Materials, Ecole Polytechnique Fédérale de Lausanne, 1015 Lausanne, Switzerland; 3 Department of Civil Engineering, Tsinghua University, Beijing 100084, China; hxc23@mails.tsinghua.edu.cn; 4 State Key Laboratory of Clean Energy Utilization, Zhejiang University, Hangzhou 310027, China; xiyan991112@163.com; 5 School of Civil Engineering, Harbin Institute of Technology, Harbin 150090, China; 6 College of Materials Science and Engineering, Chongqing University, Chongqing 400045, China; 7 School of Civil Engineering, Southeast University, Nanjing 211189, China; mbist17760258359zz@163.com; 8 Department of Civil and Environmental Engineering, The Hong Kong Polytechnic University, Kowloon, Hong Kong, China; weichtian@polyu.edu.hk

**Keywords:** rehydration, recycled cement, high temperature, C-S-H paste, microstructure

## Abstract

The effect of elevated temperatures and subsequent rehydration on the microscopic mechanical properties and physicochemical changes of cement pastes was investigated. Cement pastes with different grades (CEM I 42.5, CEM I 52.5) and different water-to-cement ratios (0.3, 0.4) were exposed to target temperatures of 300 °C, 600 °C, and 900 °C, followed by rehydration. Several characterization techniques, including the Vickers microhardness test, X-ray diffraction, thermogravimetry, and ^1^H Nuclear Magnetic Resonance spectroscopy, were employed to assess changes in the microscopic mechanical and physicochemical properties of the cement pastes resulting from the heating and rehydration treatments. The results indicate that the cement pastes with higher grades and a higher water-to-cement ratio exhibit better resistance to high temperatures. The heating process alters the water distribution and structure of C-S-H gel, leading to the collapse of its interlayer structure and an increase in gel porosity. Elevated temperatures (300 °C and 600 °C), followed by rehydration, enhance the Vickers microhardness of the cement pastes. However, excessively high temperatures (900 °C) weaken the micro-mechanical properties and may cause damage. Cement pastes heated to 600 °C show a more significant recovery in micro-mechanical properties compared to those heated at 300 °C, which is attributed to the rehydration of a new amorphous nesosilicate phase formed at 600 °C.

## 1. Introduction

Concrete is the most ubiquitous man-made material in the world and is widely used in various building structures, such as civil buildings, tunnels, and nuclear protection structures. In some cases (e.g., fire, etc.) or special structures (e.g., the interior of industrial chimneys, nuclear waste storage rooms in nuclear power plants, etc.), concrete may undergo damage and cracking due to high temperatures, which ultimately leads to a reduction in the load-bearing capacity, decreased durability, and even the collapse of the building [1,2,3]. Thus, it is of utmost importance to study the ability of concrete to resist high temperatures. The primary chemical process responsible for the mechanical deterioration of concrete is the alteration of calcium silicate hydrate (C-S-H) and other hydration products in cement paste [4,5], since cement paste serves as the most critical component in concrete, acting as a binding glue between aggregates.

The cement hydration products include calcium hydroxide (CH), C-S-H, ettringite (AFt), monosulfoaluminate, hemicarboaluminate, monocarboaluminate, and others. When temperature increases, cement hydration products gradually undergo dehydration and decomposition reactions. Thermally activated dehydrated cement usually contains a higher amount of calcium oxide and lacks C_3_S compared to ordinary silicate cement [6]. The specific composition of the dehydrated phases is mainly determined by the target temperature and the precursor material [7]. Wang et al. [8] determined the form of sustained dehydration of C-S-H over a certain range of temperatures. It was found that tobermorite gradually loses its layer water as the temperature increases, and the spacing between the layers decreases from 1.2 nm (120 °C) to 0.96 nm (450 °C), the latter corresponding to the complete removal of water from the interlayer space. In addition, the jennite becomes increasingly disordered when the temperature reaches 450 °C, while wollastonite and a small amount of C_2_S appear at 750 °C, which are less reactive than the dehydrated jennite. Furthermore, Zhang et al. [9] investigated the changes in C-S-H nanostructures at different exposure temperatures. The spherical structure of C-S-H remained mainly unchanged from 105 to 400 °C. However, at 500~700 °C, the volume of C-S-H changed significantly, with the spherical structure transforming into small C_2_S crystal particles, which merged into large particles at the temperature of 800~1000 °C. In addition to C-S-H gels, many researchers have studied the dehydration process of other cement hydration products. Cao [10] analyzed the dehydration properties and structural changes of CH and AFm in hydrated cement pastes. At 300 °C, there was no change in the morphology of CH, and the main layer structure of the AFm was dehydrated, with a weight loss of about 37%. At 500 °C, a large amount of CaO appeared, accompanied by the nearly complete disappearance of CH lamellar microcrystals, and the AFm lost all its water and decomposed to produce C_12_A_7_, CaO, and CaSO_4_. Above 700 °C, the contacts of the original network structure were greatly reduced, and the amount of CH was very small, with most of it being a partially or completely decomposed CH residue. At about 900 °C, calcium aluminate hydrate formed C_12_A_7_. At 1000 °C, dehydrated calcium sulphoaluminate (C_4_A_3_S) was produced, with both CaO and CaSO_4_ present.

The dehydrated products resulting from thermal decomposition exist as gels and have the capacity to undergo hydration reactions to form CH, C-S-H, AFt, etc., when in contact with water. Xuan and Shui [11] employed X-ray diffraction to identify the main crystalline phases in the recycled cement paste, which was previously thermally activated at 800 °C. The diffraction peaks observed were in similar positions to those of the hardened OPC, corresponding to the presence of C_2_S, CH, CaCO_3_, and C_4_AF phases. However, the peaks were broader and less intense. Their SEM analysis [11] further elucidated that the samples treated at 400 °C showed a loose microstructure of fine bundles of C-S-H surrounded by acicular calcite. However, the porosity decreased with increasing temperature, and a denser microstructure was obtained at 800 °C. The microstructure of the recycled cement paste was also found to be more compact [11]. Overall, the surface morphology of the recycled cement rehydration products was rough and irregular, which was attributed to the shorter setting time and the intense competition of the rehydration products for internal space in the microstructure.

Vyšvaril et al. [12] found that the recycled cement paste, which was previously thermally activated at 600 °C, contained predominantly CH, CaCO_3_, C_2_S, and C_4_AF. In contrast to Xuan and Shui, samples treated at 800 °C did not show hydration peaks on rehydration, whereas CA_2_S_2_H_4_ was detected above 600 °C. Wang et al. [8] indicated that the rehydrated samples showed a higher CaCO_3_ content compared to the OPC hydration products. The rehydrated products were analyzed by SEM-EDS, which exhibited a different morphology from the original hydrated cement paste, including abundant nanoscale particles that were identified as a mixture of C-S-H gel and CaCO_3_ [8]. XRD and SEM-EDS also provided evidence of calcium carbon–aluminate formation, induced by the reaction between CaCO_3_ and the aluminate phase [8]. Zhang et al. [13] described the rehydration reaction of the recycled cement thermally activated at 600 °C in three parts: the rehydration of partially dehydrated C-S-H and β-C_2_S to form C-S-H; the hydration of CaO to form CH; and the hydration of the dehydrated aluminate phases.

Overall, most studies on rehydration of thermally activated recycled cement report the rehydration of C-S-H and the presence of calcite and other hydrated aluminate phases [14,15,16,17,18,19]. Carbonated phases are frequently present and may be the result of the carbonation of the dehydrated cement during the heating process. The rehydration products are related to the thermal activation temperature and more systematic studies are needed to characterize the rehydrated phases in thermally activated cement pastes.

Numerous investigations have been carried out to explore the impact of rehydration on the mechanical and durability properties of cementitious materials [20,21,22,23,24,25,26]. This phenomenon stems from the common exposure of fire-damaged concrete to water during extinguishment, making it significantly meaningful to employ effective rehydration techniques for the restoration of the damaged concrete [4,11,14,27,28]. It was found that when cement pastes underwent a 28-day rehydration after heating to 200 °C and 400 °C, the compressive strength of the pastes could be enhanced as a result of the hydration of residual anhydrous cement clinker [27]. Additionally, it was also found that the rehydration process could promote the recovery of cracks caused by high temperatures [4]. On the contrary, the strength of samples heated at temperatures above 600 °C decreased after the rehydration, which was attributed to the decomposition of the paste [27].

Understanding the mechanical recycling properties of cementitious materials damaged by high temperatures is crucial for assessing the reusability of concrete buildings exposed to high temperatures (e.g., fires). However, the details of the recycling mechanisms for rehydration under a variety of different initial conditions (different strength classes, different water–cement ratios) have not yet been fully elucidated. Thus, we investigated the effects of precursor materials with different strength levels (CEM I 42.5, CEM I 52.5) and water-to-cement ratios (0.3, 0.4) on the mechanical and structural recovery of Portland cement paste after being subjected to three different target temperatures (300, 600, and 900 °C). The variations in the microscopic strength and surface morphology of the pastes due to heating and rehydration were characterized. Moreover, the detailed process of the formation and decomposition of hydration products in the pastes due to the heating and rehydration were analyzed using XRD, TG, and ^1^H NMR.

## 2. Experimental Program

### 2.1. Materials and Sample Preparation

In this study, two types of white cement (CEM I 42.5, CEM I 52.5) were used (provided by Aalborg Portland (Anqing) Co., Ltd., Anqing, China), and their chemical compositions are given in Table 1. The white cement paste was produced with water at water-to-cement ratios of 0.4 and 0.3 as cylinder samples of Φ20 × 25 mm. The NJ-160A cement paste mixer was used. The mixer was set to run at a low speed (rotation 140 ± 5 r/min, revolution 62 ± 5 r/min) for 90 s, stop for 15 s to scrape off the paste adhered to the blade and the pot wall, and then run at a high speed (rotation 285 ± 10 r/min, revolution 125 ± 10 r/min) for 90 s before casting. The cylinder samples are denoted as Groups 4-4, 5-4, and 5-3, where the first number refers to the strength level and the latter refers to the water-to-cement ratio. The cement mixtures were subjected to a standard curing (20 ± 2 °C and over 95% RH) lasting for 2 years to obtain a complete hydration. Then, the cylinders were exposed to heat treatment with three different target temperatures (300, 600, and 900 °C) to simulate different fire damage or elevated temperature environments. The heated samples were named by adding the temperature value to the original label of the cement pastes, e.g., 4-4-300 is read as 4-4 cement paste with a target temperature of 300 °C. The heating procedure consisted of a rapid heating stage with a rate of 10 °C/min, a stable stage at the target temperature for 3 h, and a natural cooling stage until room temperature was reached (SX2-5-12 Muffle furnace was used). Afterwards, the undamaged samples were chosen to be subjected to a water-rehydration stage for 2 days. The rehydrated samples were named by adding “R” to the label of the heated samples, e.g., changing 4-4-300 to R-4-4-300. Due to the non-destructive characteristics of ^1^H Nuclear Magnetic Resonance spectroscopy (^1^H NMR), each sample underwent ^1^H NMR tests at three time points: before the heating treatment, after the heating treatment, and after rehydration. In addition, the parallel unheated samples, heated samples, and rehydration samples were characterized using the Vickers Microhardness test, X-ray diffraction, and thermogravimetry.

### 2.2. Characterization Techniques

The Vickers Microhardness test: Microhardness indentation testing was performed using a digital Vickers microhardness tester (HVX-1000A, Shenzhen, China). The load and holding time were set as 300 g and 10 s, respectively, during the loading process for each test. A grid of 3 × 3 was adopted in each sample, with 9 indentation points. The spacing between each point was 100 μm.

X-ray diffraction: X-ray diffraction was used to determine the crystalline phases of samples. The tests were conducted by a D/max-2550 X-ray diffractometer (Rigaku, Akishima, Japan), which operated at 40 kV and 40 mA with CuK_α_ radiation (λ = 1.5418 Å). The powdery samples underwent the scanning within the 5–65° 2*θ* range, employing a step size of 0.02° and a rate of 0.6 s per step.

Thermogravimetry: The STA 449 F3 (Netzsh, Selb, Germany) synchronous thermal analyzer was utilized. Approximately 50 mg of each sample was heated from 50 to 1000 °C at a rate of 10 °C/min in an alumina crucible. The heating process was under an argon atmosphere flow (60 mL/min).

The ^1^H Nuclear Magnetic Resonance spectroscopy: The ^1^H NMR measurements were performed on a VTMR20-010V-I spectrometer manufactured by NIUMAG in Suzhou, China, operating at a magnetic field strength of 0.5 ± 0.05 T. The spectrometer generates a field homogeneity deviation of less than 30 ppm and a field stability with a drift rate of less than 200 Hz per hour. The instrument’s main frequency is approximately 21 MHz, and it utilizes a probe coil with a diameter of 40 mm. The dead time and the 90° pulse length of the system are 13 μs and 2.8 μs, respectively. The relaxation signal collected was inverted using the InvFit inversion program to obtain the transverse relaxation time (T_2_) data of the samples [29].

## 3. Results and Analyses

### 3.1. Macro–Meso–Micro Changes of Cement Pastes Subjected to Elevated Temperatures

**Figure 1:**
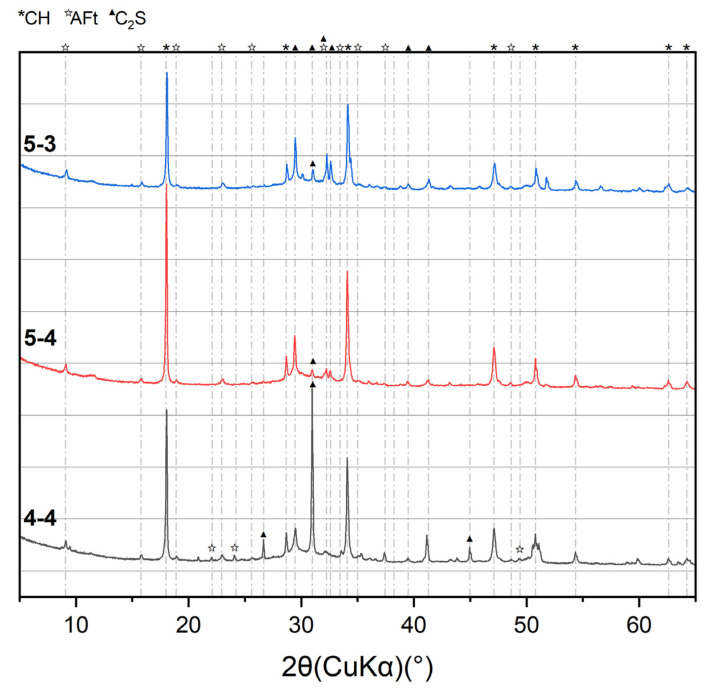
XRD results of the original samples.

**Figure 2:**
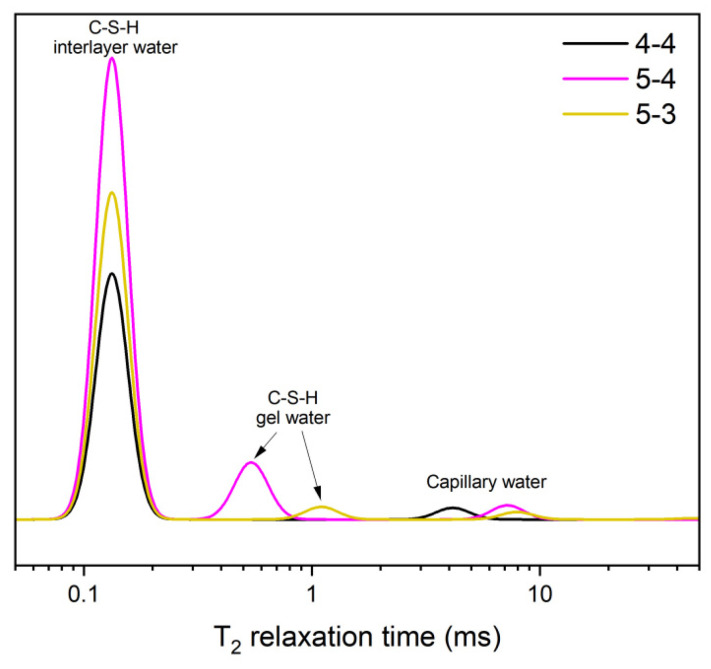
The ^1^H NMR results of the original samples.

Figure 1 presents the XRD patterns of the cylinders before the heating treatment. In addition to the common hydrations, such as portlandite (CH) and ettringte (AFt), dicalcium silicate (C_2_S) was also detected in the XRD results for three cement cylinders. The lower intensity of the CH peak and the higher intensity of the C_2_S peak in Group 5-3 than those in the other two groups indicates a lower hydration degree due to the small water-to-cement ratio, which is consistent with existing research [5]. Figure 2 presents the T_2_ distribution of the samples before the heating treatment via ^1^H NMR tests. According to [30,31], three peaks, approximately 0.1 ms, 0.1–1 ms, and 10 ms, correspond to the C-S-H interlayer water, C-S-H gel water, and capillary water, respectively. In terms of the C-S-H interlayer and gel pore value, both present the order of 5-4 > 5-3 > 4-4. This sequence also demonstrates the order of the C-S-H content formed during the hydration process. The largest C-S-H content in Group 5-4 is contributed to the large original C_3_S content in Table 1 and the high water-to-cement ratio. The smallest C-S-H content in 4-4 originates from the low C_3_S content.

**Figure 3:**
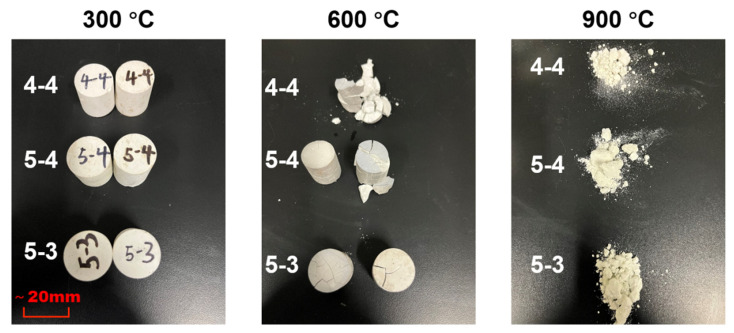
Morphology of samples after heating at 300 °C, 600 °C, and 900 °C. (The lengths may deviate slightly due to the angle at which the photos were taken).

Figure 3 displays the appearance of the samples after the heating treatment. No conspicuous cracks were observed in any of the samples subjected to the temperature of 300 °C. When the temperature reached 600 °C, all the samples cracked, and they even broke into pieces in Group 4-4. The samples were fragmented into powders, resulting in full disintegration. The aggressive effect of the high temperature can be attributed to two factors: firstly, the increase in capillary pressure due to the drop in RH with the elevated temperature; secondly, the loss of the adhesion of the C-S-H gels owing to the change in their microstructure. The latter will be discussed hereafter.

**Figure 4:**
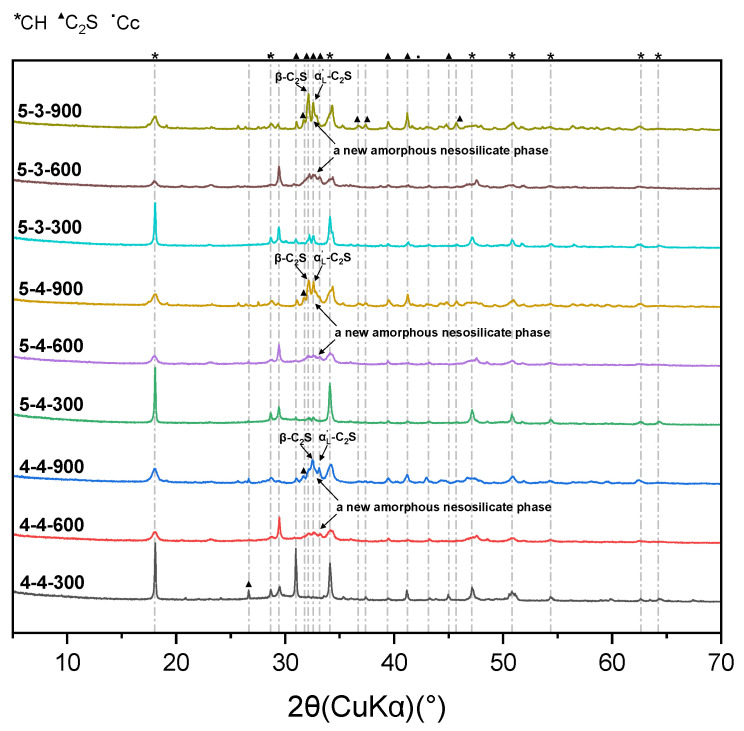
XRD results of samples heated at 300 °C, 600 °C, and 900 °C.

Figure 4 displays the XRD results of the heated samples. As shown in Figure 4, the reflection peaked at 18.02°, corresponding to CH, and exhibited a decrease as the heating temperature rose from 300 °C to 600 °C, indicating that CH remained stable at around 300 °C but underwent decomposition at 600 °C, which coincided with its decomposition temperature range (400–500 °C). Conversely, the reflection peaks at 29.42°, corresponding to CaCO_3_, initially exhibited an increase followed by a decrease, as the heating temperature increased from 300 °C to 900 °C. It suggested that at 600 °C, certain calcium-bearing solids (such as CH, C_2_S, and C-S-H) were carbonated to form CaCO_3_, which subsequently decomposed at 900 °C. Moreover, a large amount of β-C_2_S and α’L-C_2_S were detected at the heating temperatures of 600 °C and 900 °C, especially at 900 °C. The formation of C_2_S that had excellent crystallization but little hydration reactivity [7,31] can be attributed to the decomposition of the C-S-H gel over 600 °C. The newly formed C_2_S at high temperatures, especially the amorphous C_2_S at 600 °C, can provide a rehydration potential for the heated samples, which will be discussed later.

**Figure 5:**
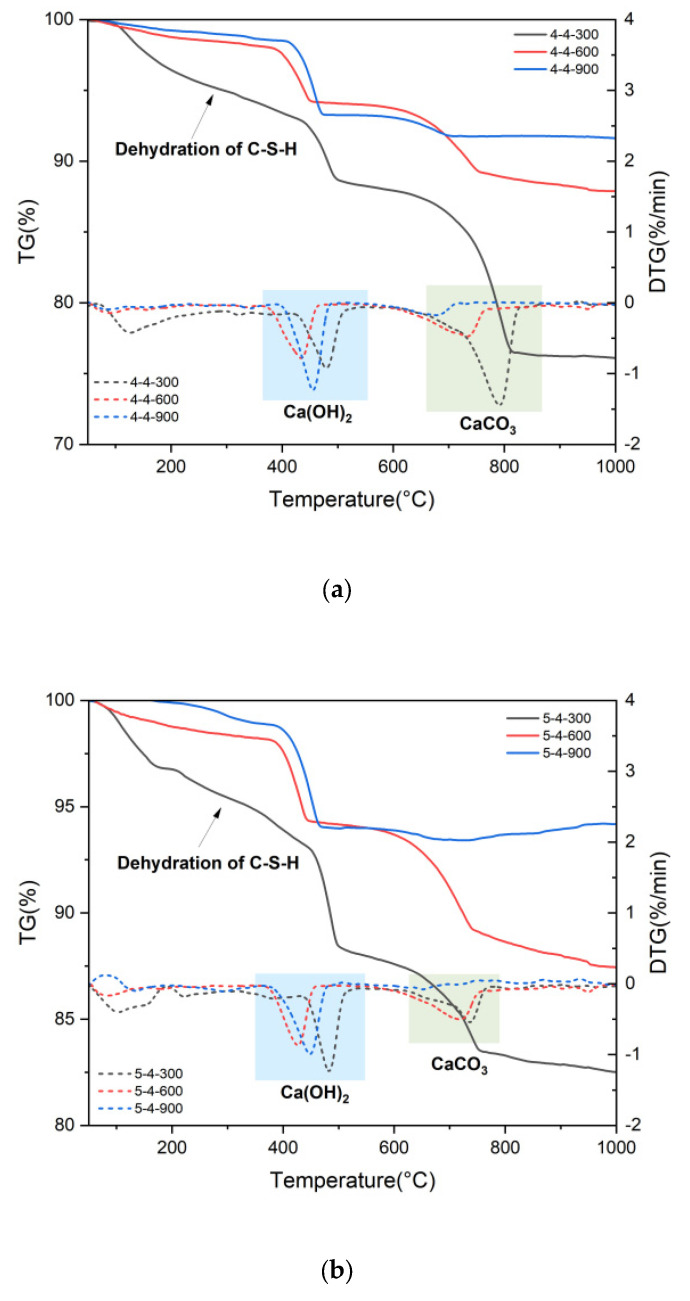

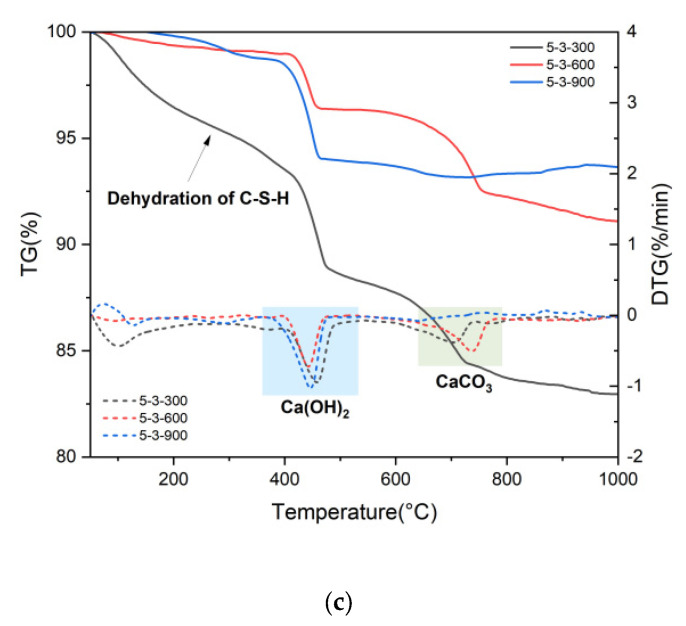
TG-DTG results of samples (**a**) 4-4, (**b**), 5-4 (**c**), 5-3 heated at 300 °C, 600 °C, and 900 °C.

**Figure 6:**
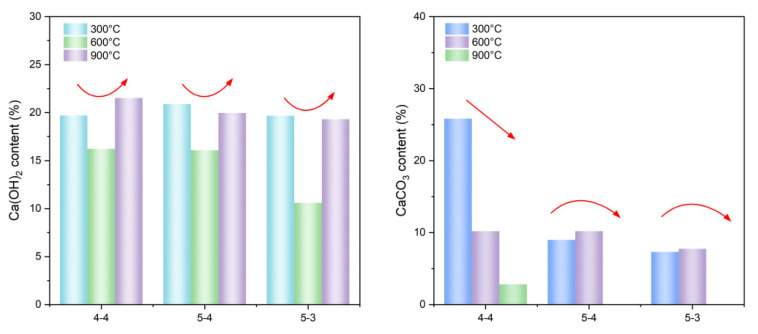
Hydration products content of samples heated at 300 °C, 600 °C, and 900 °C. (Red arrows indicate trends in Ca(OH)_2_ and CaCO_3_ content).

Figure 5 presents the TG-DTG results of heated samples. The CH and CaCO_3_ contents of heated samples were quantified using the tangential method [32] based on the TG-DTG results, shown in Figure 6. The quantification process was based on chemical Equations (1) and (2). As can be seen in Figure 5a–c, the samples heated at 300 °C all exhibited pronounced weight loss areas corresponding to the dehydration of C-S-H. In contrast, since the samples heated at 600 °C and 900 °C had already undergone significant water loss, their TG curves were more stable at the temperature interval of 50 °C to 400 °C. CH begins to decompose at around 460 °C [32]. Thus, the CH in the samples heated at 600 °C and 900 °C may mainly have come from the rehydration of CaO, caused by the uptake of atmospheric water vapor during the cooling process [33]. Additionally, newly formed CH has a lower bonding energy, consequently leading to a lower decomposition temperature [31], as shown in Figure 5. As depicted in Figure 6, the content of CH decreased from 300 °C to 600 °C, and then increased from 600 °C to 900 °C, consistent with the XRD results. The decline in the CH content from 300 °C to 600 °C resulted from its decomposition at elevated temperatures of over 400 °C. However, the decomposition of CaCO_3_ yielded CaO, which reacted with water vapor during the sample cooling, leading to the increase in the CH content from 600 °C to 900 °C. Group 4-4 exhibited a continuous decrease in the CaCO_3_ content when experiencing the elevated temperatures from 300 °C to 900 °C. Groups 5-4 and 5-3 initially displayed an increase, followed by a decrease, in the CaCO_3_ content within the same temperature range, consistent with the XRD results. The initial rise was attributed to the carbonation of the calcium-bearing phase, while the subsequent decline stemmed from the decomposition of CaCO_3_ above 600 °C.

Ca(OH)_2_ → CaO + H_2_O (1)

CaCO_3_ → CaO + CO_2_ (2)

**Figure 7:**
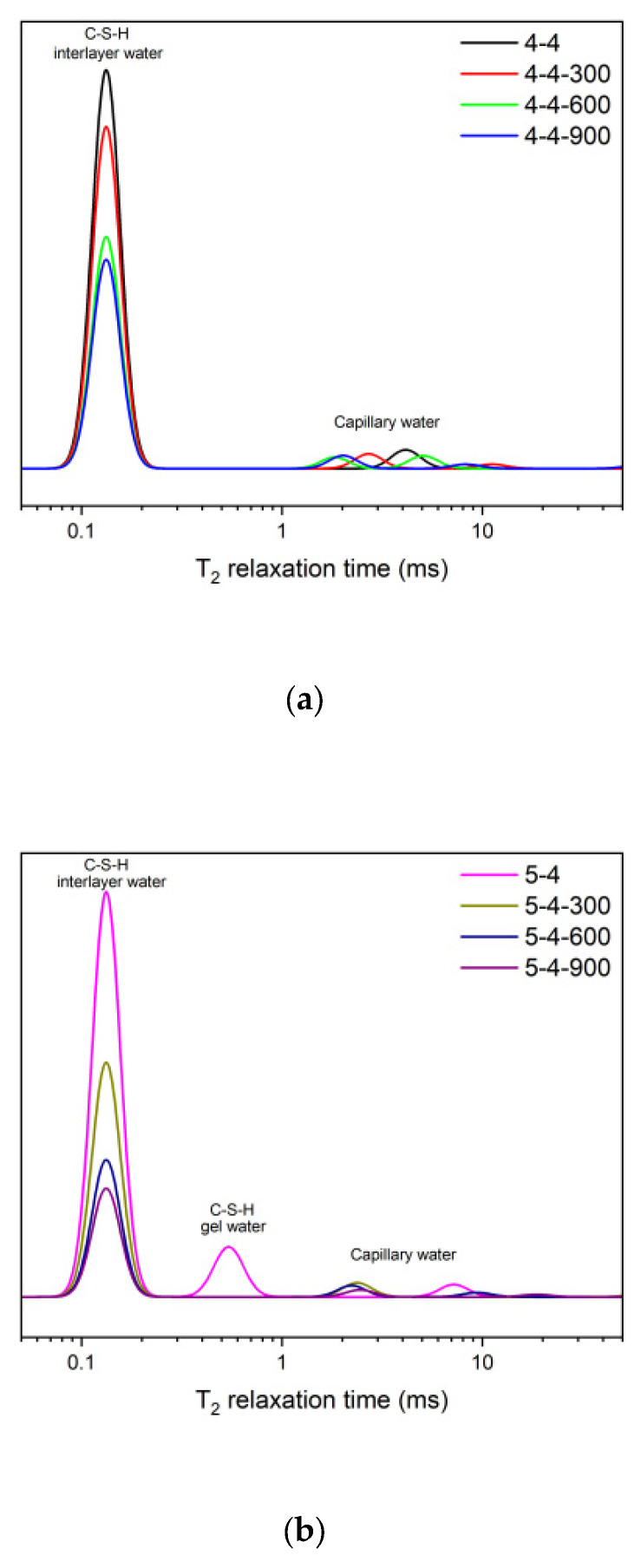

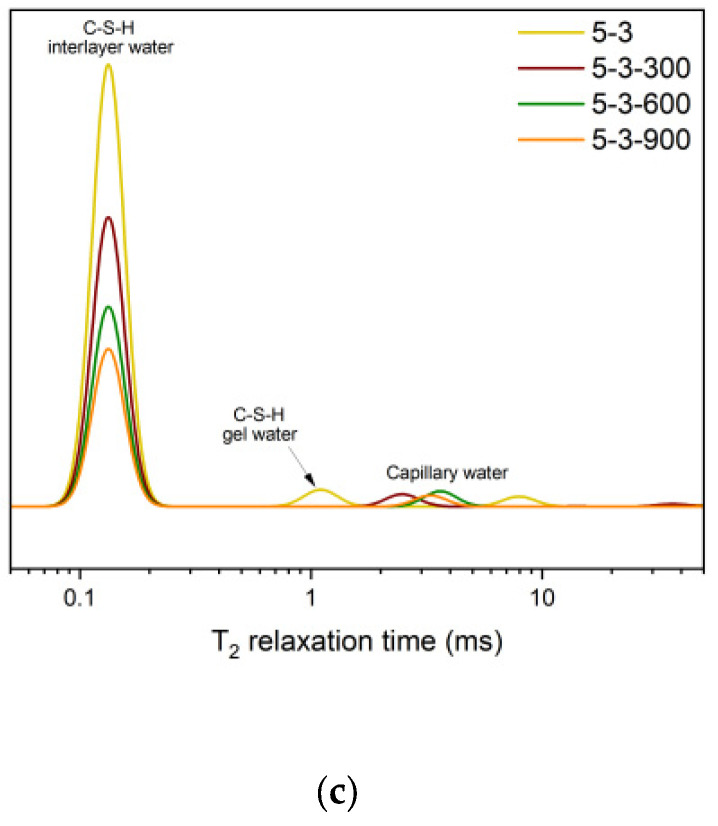
The ^1^H NMR results of the original samples (**a**) 4-4, (**b**) 5-4, (**c**) 5-3, and the samples heated at 300 °C, 600 °C, and 900 °C.

The ^1^H NMR spectra of the unheated original samples and the samples heated at 300 °C, 600 °C, and 900 °C are presented in Figure 7. The figures show a decrease in the C-S-H interlayer water with the increasing heating temperature. Specifically, a significant drop was observed in the peak corresponding to the C-S-H interlayer water from Group 4-4-300 to Group 4-4-600, Group 5-4-300 to Group 5-4-600, and Group 5-3-300 to Group 5-3-600. It suggested that a significant loss of C-S-H interlayer water occurred when the heating temperature was elevated from 300 °C to 600 °C, which could have led to a considerable change in the C-S-H structure. A similar drop was also observed from Group 4-4 to Group 4-4-300, Group 5-4 to Group 5-4-300, and Group 5-3 to Group 5-3-300. However, when the heating temperature was elevated from 600 °C to 900 °C, the loss of C-S-H interlayer water was not that notable. For Group 5-4 and Group 5-3, the C-S-H gel water was completely lost when heated to 300 °C. Furthermore, besides the change in the C-S-H interlayer water content, a noticeable leftward shift in the position of the capillary water peak was observed after the samples were heated. The relaxation time of the water signal is positively correlated with the pore size in which it is situated [30]. Therefore, the leftward shift of the capillary water peak suggested that the capillary pore size of the heated samples might become smaller, which was attributed to CaCO_3_. As shown in Figure 4, CaCO_3_ was produced after the heating process (300 °C and 600 °C), which refined the pore structure effectively. Previous research [34,35] also indicated that the CaCO_3_ produced, owing to carbonation, would precipitate in the capillary pores and could decrease the large capillary porosity.

**Figure 8:**
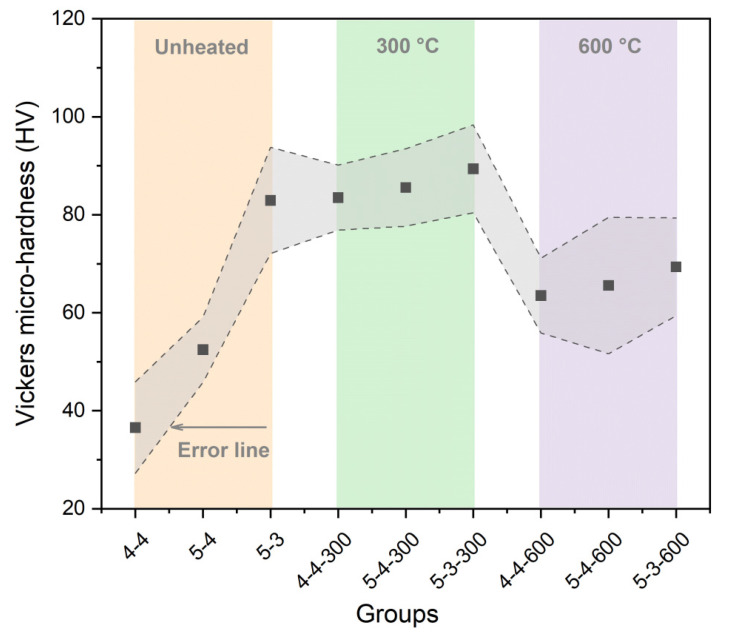
Vickers microhardness results of the original samples and the samples heated at 300 °C and 600 °C.

Figure 8 displays the Vickers microhardness of the samples heated at 300 °C, 600 °C, and the samples that were not heated. The Vickers microhardness of the samples heated at 300 °C was significantly enhanced, especially for Group 4-4 and Group 5-4, which had higher water-to-cement ratios. Before heating, the higher water-to-cement ratio led to more C-S-H interlayer water, which could act as a lubricant in the interlayer region [36,37], and the cohesion force of the interatomic bond would be subsequently weakened [36,37]. Thus, the loss of C-S-H interlayer water during the heating process at 300 °C led to the enhancement of the Vickers microhardness. However, the Vickers microhardness of the samples heated at 600 °C was lower compared to those heated at 300 °C, which was attributed to the excessive reduction in the C-S-H interlayer water, as shown in Figure 7. The excessive loss of C-S-H interlayer water would lead to the collapse of the C-S-H structure, resulting in a decrease in its mechanical properties [36]. But most of the samples heated at 600 °C still maintained a higher Vickers microhardness than the original samples. However, as in Figure 8, when the sample with the water-to-cement ratio of 0.3 was heated to 600 °C, its Vickers microhardness was even lower than the original sample (Group 5-3). Thus, the collapse of the C-S-H structure might be more likely to occur in the samples with a lower water-to-cement ratio during the heating process. Furthermore, the samples heated at 600 °C exhibited noticeable cracks, which were also detrimental to their macroscopic mechanical properties.

### 3.2. Physical and Chemical Changes of High-Temperature Dehydrated Cement Pastes After Rehydration

**Figure 9:**
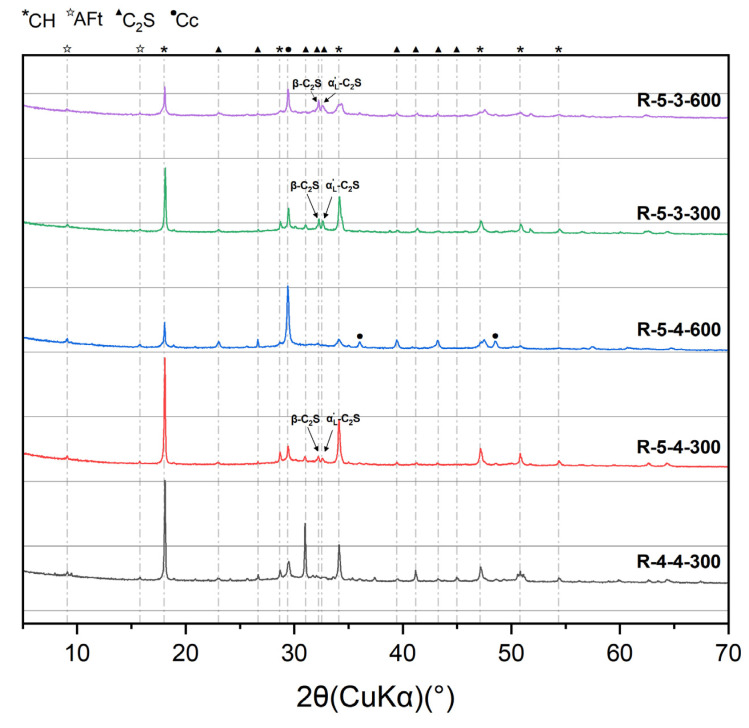
XRD results of heated samples after rehydration.

Figure 9 displays the XRD results of the heated samples after rehydration. Group 4-4-600 and the groups heated at 900 °C did not participate in the rehydration process, as they experienced a complete loss of their mechanical properties. A small amount of AFt was identified in the five groups, demonstrating that these samples had undergone rehydration reactions. Additionally, the gentle slope around 31.52° to 33.44°, corresponding to β-C_2_S, α’L-C_2_S, and a new amorphous nesosilicate phase [31], transformed into sharp peaks after rehydration. This indicated that while the new amorphous nesosilicate phase was consumed, the well-crystallized β-C_2_S and α’L-C_2_S remained stable.

**Figure 10:**
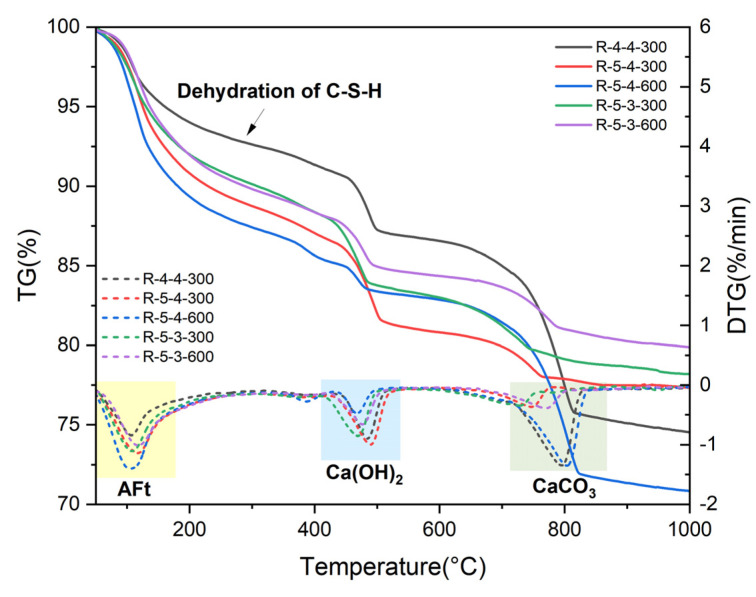
TG-DTG results of heated samples after rehydration.

Figure 10 presents the TG-DTG results of the rehydrated samples. As can be seen in the diagram, all the samples exhibited obvious weight loss areas corresponding to the dehydration of C-S-H. The rehydration product AFt was identified, shown as the peaks at around 100 °C. The TG-DTG results also confirmed the rehydration of C-S-H and other anhydrous phases.

**Figure 11:**
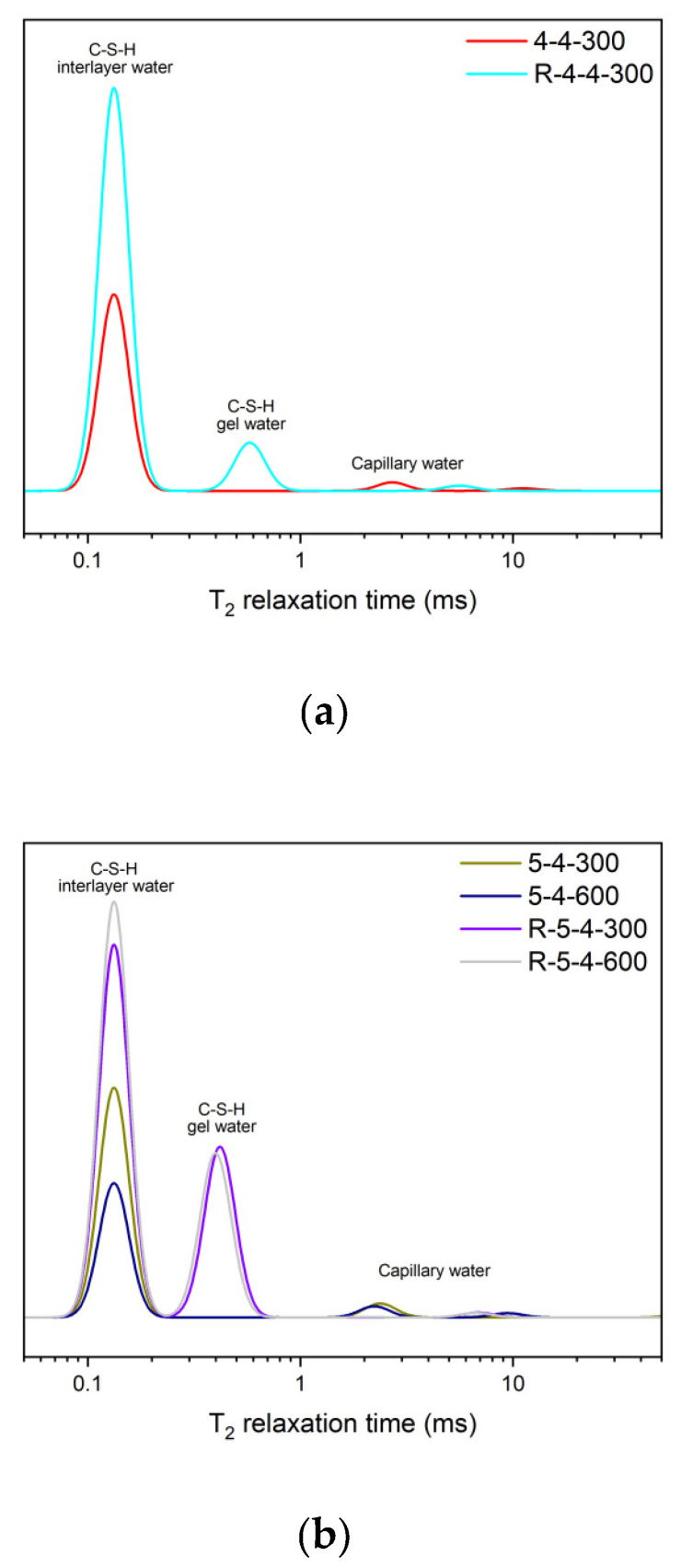

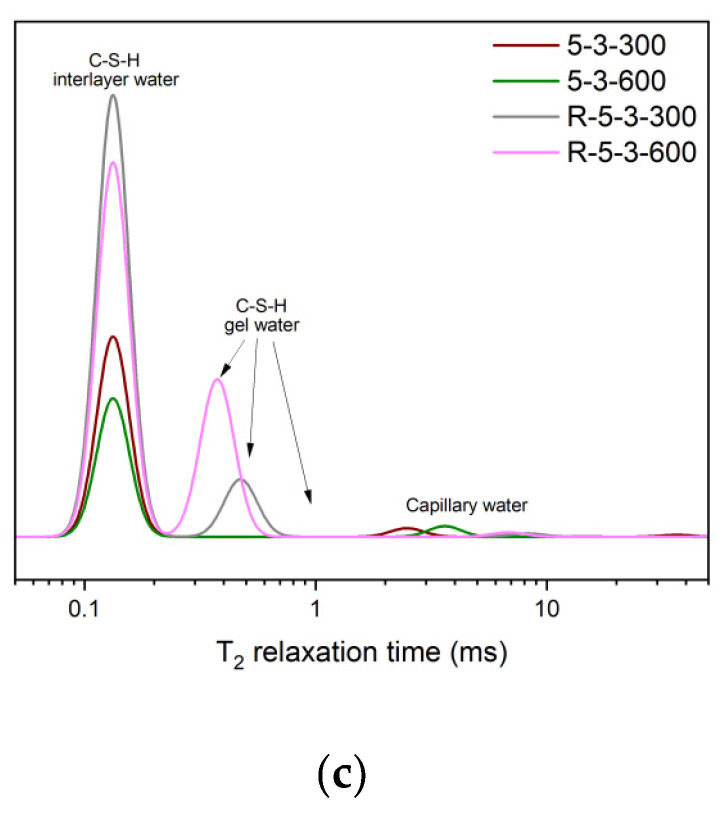
The ^1^H NMR results of the heated samples (**a**) 4-4, (**b**) 5-4, (**c**) 5-3, after rehydration.

The ^1^H NMR spectra of the rehydrated samples are displayed in Figure 11. The results demonstrated that the heated samples had regained a large quantity of C-S-H interlayer water after rehydration. The amount of C-S-H interlayer water in the rehydrated samples was even higher than that of the unheated original samples. Additionally, C-S-H gel water was also detected in the rehydrated samples, which was absent in the heated samples. The results suggested that the dehydrated C-S-H would restore its original gel microstructures and mechanical properties when subjected to water again, which can be further verified by the results of the Vickers microhardness analysis in the following paragraphs.

**Figure 12:**
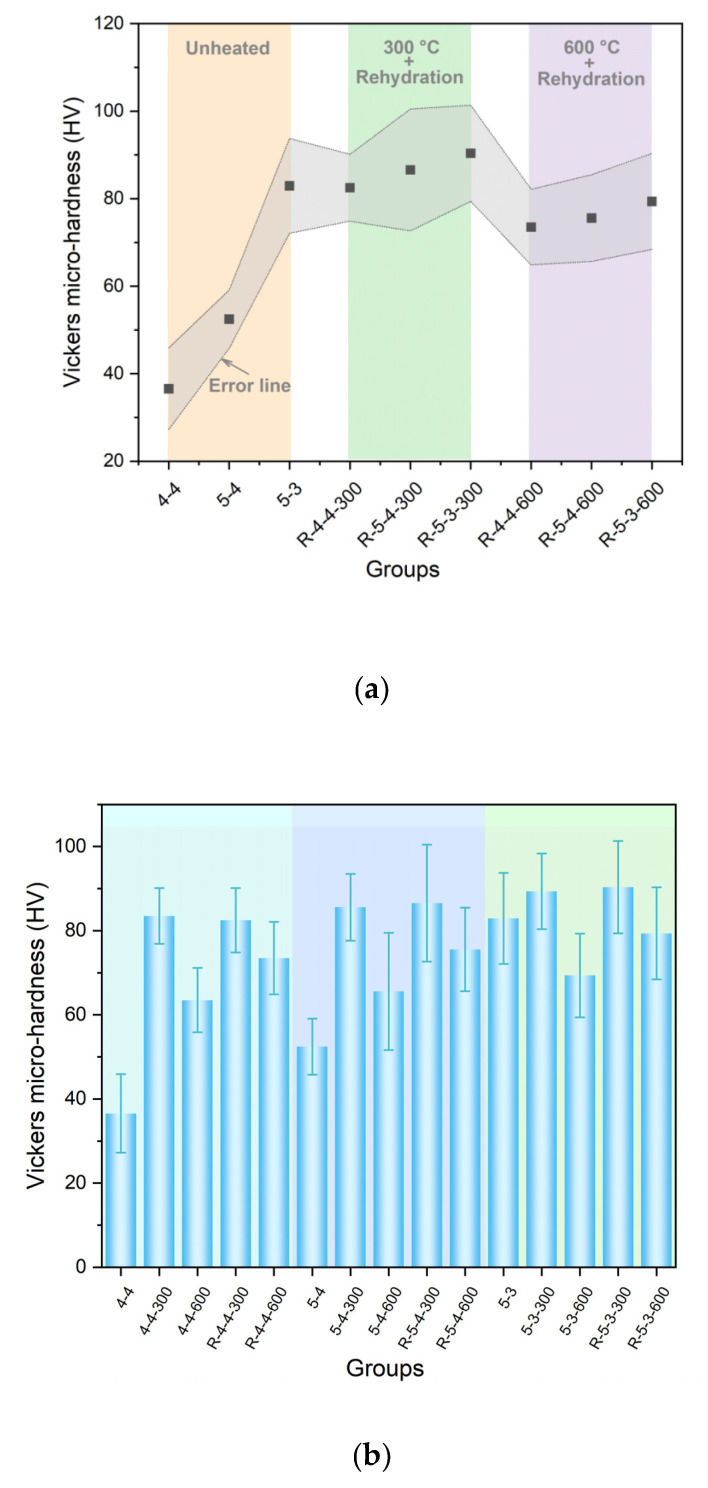
Vickers microhardness results of (**a**) original samples and rehydrated samples and (**b**) all samples except for damaged samples.

Figure 12 exhibits the difference in the Vickers microhardness among the original samples, heated samples, and rehydrated samples. As can be seen in Figure 12a, after rehydration, the Vickers microhardness of the samples heated at 600 °C approached that of the samples heated at 300 °C. However, as displayed above in Figure 8, before rehydration, the Vickers microhardness of the samples heated at 600 °C was significantly lower than that of the samples heated at 300 °C, with a difference of approximately 20 HV. Additionally, as displayed in Figure 12b, the samples heated at 600 °C exhibited a greater micro-mechanical property gain after rehydration, compared with the samples heated at 300 °C. It suggested that rehydration significantly promoted the mechanical recovery of the samples heated at 600 °C. The effect of rehydration was more significant in the samples heated at 600 °C, compared to the samples heated at 300 °C. This phenomenon was attributed to the formation of more C_2_S and the new amorphous nesosilicate phase with hydration reactivity in the samples heated at 600 °C, according to the XRD results shown in Figure 4. Moreover, as shown in Figure 3, the samples heated at 600 °C exhibited noticeable cracks, which was conducive for water permeation and consequently facilitated the rehydration process.

## 4. Discussion

**Figure 13:**
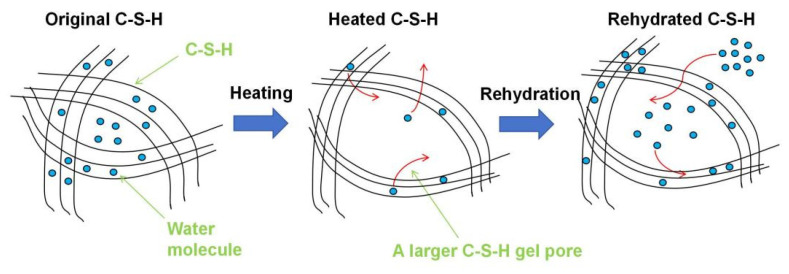
Evolution of C-S-H structure and moisture migration during heating and rehydration process. (Red arrows indicate the direction of moisture migration).

During the heating process, water within C-S-H migrates from the C-S-H interlayer spaces to the C-S-H gel pores and further to the larger pores, eventually being removed through drying. The loss of the C-S-H interlayer water results in a reduction in interlayer spacing and can eventually lead to the collapse of the C-S-H interlayer structure. The collapse of the C-S-H interlayer structure consequently leads to an enlargement of the C-S-H gel pores. Subsequently, while undergoing the rehydration process, external water re-enters the C-S-H gel pores, with a portion remaining within the gel pores and another portion re-entering the interlayer spaces of C-S-H. Due to the enlargement of the C-S-H gel pores, there is a significant increase in the content of C-S-H gel water after rehydration, as evidenced by the ^1^H NMR results in Figure 11. The evolution of the C-S-H structure and moisture migration during the heating–rehydration process is depicted in Figure 13, which is similar to the C-S-H structure rearrangement phenomenon [30,38].

Elevated temperatures have a significant impact on the mechanical properties and rehydration characteristics of cement pastes. The heating process can reduce the content of C-S-H interlayer water in cement pastes, as shown in Figure 8. Before the collapse of C-S-H, the reduction in C-S-H interlayer water can improve its microstrength [36,37]; however, the excessive loss of C-S-H interlayer water will lead to the collapse of the C-S-H structure and a significant decrease in its microstrength. Therefore, the Vickers microhardness of the samples heated at 300 °C surpassed that of the samples that were not heated, while the Vickers microhardness of the samples heated at 600 °C exceeded that of the samples that were not heated but remained lower than that of the samples heated at 300 °C, as shown in Figure 12b. Additionally, the CaCO_3_ generated after heating process can also improve the microstrength of the pastes by refining their pore structure, according to the results of the XRD and ^1^H NMR shown in Figure 4 and Figure 7. Thus, it can be concluded that elevated temperatures can enhance the microstrength of cement pastes. However, excessively high temperatures diminish this strength-enhancing effect and may result in cracking and even the complete destruction of the cement pastes.

According to the results of the XRD in Figure 4, elevated temperatures lead to the formation of β-C_2_S and α’L-C_2_S with excellent crystallization but little hydration reactivity. But the new amorphous nesosilicate phase generated at the heating temperature of 600 °C can promote the rehydration process, which consequently results in a more significant enhancement of microstrength, compared with the heating temperature of 300 °C. Therefore, a higher heating temperature (600 °C in this paper) leads to a more excellent rehydration capacity in cement pastes.

**Figure 14:**
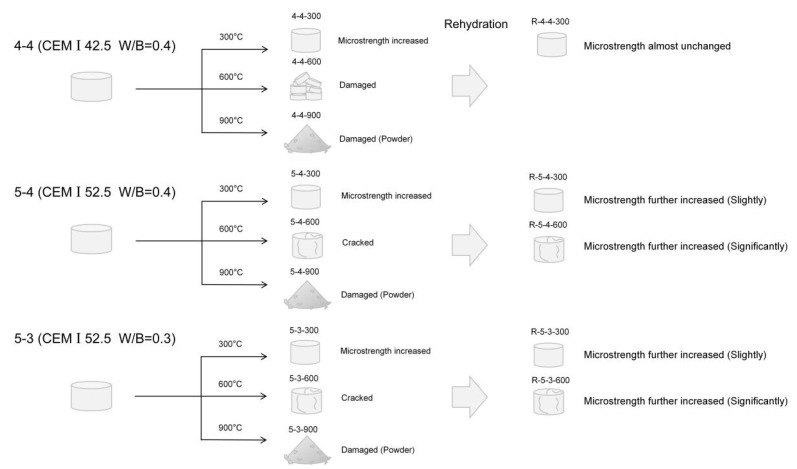
Impact of different high temperatures on different samples.

In terms of the water-to-cement ratio, samples with a higher ratio (0.4) exhibited a greater increase in the Vickers microhardness compared to the samples with a lower ratio (0.3) when subjected to the same heating temperature treatment. Since the samples with a lower water-to-cement ratio have less C-S-H interlayer water, the collapse of the C-S-H structure may first occur in the samples with a lower ratio during the heating process. Regarding the cement types, the samples produced from CEM I 42.5 had already been severely damaged when they were heated to 600 °C, while samples produced from CEM I 52.5 only exhibited some cracks. Additionally, although the Vickers microhardness of Group 4-4 was significantly lower than that of Group 5-4, the hardness of the two groups became very close to each other after the high-temperature treatment. It can be attributed to the fact that the high-temperature treatment promoted the hydration of the sample of Group 4-4. In summary, the impact of high temperatures on different samples is elucidated in Figure 14.

## 5. Conclusions

The results of this study provide a comprehensive and better understanding of the impact of elevated temperatures on the mechanical properties and rehydration characteristics of cement pastes derived from different cement types with various water-to-cement ratios. The key findings are summarized as follows:

(1) Elevated temperatures significantly affect the structure and water distribution of C-S-H. During the heating process, the C-S-H interlayer water enters the C-S-H gel pores, subsequently migrates outward from the gel pores, and is eventually removed. This phenomenon leads to the structural rearrangement of C-S-H, which involves the collapse of the interlayer structure of C-S-H and the enlargement of the C-S-H gel pores.
(2) Elevated temperatures can enhance the Vickers microhardness of cement pastes by removing the C-S-H interlayer water. Additionally, carbonation due to high-temperature (300 °C and 600 °C in this paper) treatment also increases the Vickers microhardness of cement pastes by refining their pore structures. However, excessively high temperatures (900 °C) and excessive interlayer water losses can lead to the collapse of the C-S-H structure, thereby weakening the Vickers microhardness of cement pastes and potentially leading to damage.
(3) Elevated temperatures lead to the formation of well-crystallized β-C_2_S and α’L-C_2_S with little hydration reactivity. Compared to samples heated at 300 °C, those heated at 600 °C demonstrate the more significant recovery of the micro-mechanical properties. This phenomenon is attributed to the rehydration of a new amorphous nesosilicate phase that is generated at 600 °C.
(4) Samples with a lower water-to-cement ratio contain less C-S-H interlayer water, making the collapse of the C-S-H structure more likely to occur in these samples. Moreover, samples made from a high grade of cement exhibit better resistance to high temperatures. However, high-temperature treatment promotes cement hydration, thus eliminating the differences in the Vickers microhardness due to the variations in cement types to some extent.

## Figures and Tables

**Table 1:** Chemical compositions of two kinds of white cement (wt. %).

Oxide/Element	CaO	SiO_2_	SO_3_	Al_2_O_3_	MgO	K_2_O	Na_2_O	Fe_2_O_3_	TiO_2_	SrO	P_2_O_5_	MnO	NiO	CuO	Cl	Others	LOI
CEM I 42.5	60.990	19.838	4.535	2.417	9.726	0.697	0.375	0.310	0.053	0.035	0.048	0.028	0.016	0.015	0.046	-	0.872
CEM I 52.5	78.305	13.400	2.841	2.156	1.321	0.411	0.305	0.233	0.150	0.072	0.019	0.010	0.009	0.008	0.003	0.091	0.666

## Data Availability

The original contributions presented in the study are included in the article, further inquiries can be directed to the corresponding author.
